# Equality, diversity, and inclusion in oncology clinical trials: an audit of essential documents and data collection against INCLUDE under-served groups in a UK academic trial setting

**DOI:** 10.1186/s12910-023-00987-w

**Published:** 2023-11-28

**Authors:** Dhrusti Patel, Lucy Kilburn, Lisa Fox, Emma Hall, Judith Bliss, Rebecca Lewis

**Affiliations:** Clinical Trials and Statistics Unit, The Institute of Cancer Research, London, UK

**Keywords:** Oncology clinical trials, Equality, Inclusivity, Diversity, Data collection, NIHR INCLUDE framework

## Abstract

**Background:**

Clinical trials should be as inclusive as possible to facilitate equitable access to research and better reflect the population towards which any intervention is aimed. Informed by the UK’s National Institute for Health and Care Research (NIHR) Innovations in Clinical Trial Design and Delivery for the Under-served (INCLUDE) guidance, we audited oncology trials conducted by the Clinical Trials and Statistics Unit at The Institute of Cancer Research, London (ICR-CTSU) to identify whether essential documents were overtly excluding any groups and whether sufficient data were collected to assess diversity of trial participants from groups suggested by INCLUDE as under-served by research in the UK.

**Methods:**

Thirty cancer clinical trials managed by ICR-CTSU and approved between 2011–2021 were audited. The first ethics approved version of each trial’s protocol, patient information sheet, and patient completed questionnaire, together with the first case report forms (CRFs) version were reviewed. A range of items aligned with the INCLUDE under-served groups were assessed, including age, sex and gender, socio-economic and health factors. The scope did not cover trial processes in participating hospitals.

**Results:**

Data relating to participants’ age, ethnic group and health status were well collected and no upper age limit was specified in any trials’ eligibility criteria. 23/30 (77%) information sheets used at least one gendered term to address patients. Most CRFs did not specify whether they were collecting sex or gender and only included male or female categories. The median reading age for information sheets was 15–16 years (IQR: 14–15 – 16–17). Socio-economic factors were not routinely collected and not commonly mentioned in trial protocols.

**Conclusions:**

No systemic issues were identified in protocols which would explicitly prevent any under-served group from participating. Areas for improvement include reducing use of gendered words and improving readability of patient information. The challenge of fully assessing adequate inclusion of under-served populations remains, as socio-economic factors are not routinely collected because they fall beyond the data generally required for protocol-specified trial endpoint assessments. This audit has highlighted the need to agree and standardise demographic data collection to permit adequate monitoring of the under-served groups identified by the NIHR.

## Background

Clinical trials should be as inclusive as possible to allow robust assessment of the utility, efficacy, and safety of an intervention in a sample representative of the population who may ultimately receive it. This is critical in oncology trials, both to aid generalisability of results and from an equity and accessibility perspective as they can provide access to alternative, though unproven, treatment options when all other treatment has failed.

Whilst the US’ National Institutes of Health (NIH) implemented guidelines in 1990 regarding inclusion of women and minority groups in NIH supported research, [1] the focus of UK funders has been largely on equality in terms of those applying for funding, rather than those participating. However the importance of inclusivity was emphasised in the most recent strategy of the UK’s NHS Health Research Authority, the body responsible for ethics review of research involving the NHS [2].

One of the largest UK funders of non-commercial research, the National Institute for Health and Care Research (NIHR), recently released its first Equality, Diversity and Inclusion Strategy (2022–2027).[3] This includes objectives around improved tracking and reporting of diversity of research participants, focusing on protected characteristics according to the UK’s Equality Act 2010 legislation (age, disability, gender reassignment, marriage and civil partnership, pregnancy and maternity, race, religion or belief, sex, sexual orientation). The NIHR also incorporate other groups of interest consistent with those identified by their ‘Innovations in Clinical Trial Design and Delivery for the Under-served’ (INCLUDE) project [4]. Whilst INCLUDE does not provide one single definition of a universally under-served group in the UK, they note that key characteristics common to under-served groups are: lower inclusion in research than expected from population estimates, high disease burden unmatched by the volume of research, and differences in how some groups may engage with healthcare interventions without research into these differences. Examples of potentially under-served groups are essentially anyone outside the white male archetype traditionally considered a ‘standard human’ in medical research, which historically aimed to limit heterogeneity amongst participants to try and generate consistent results [5]. Suggested INCLUDE groups are intersectional and may comprise people from less represented biological sexes, genders, ethnic groups, age extremes, people with underlying health conditions and disabilities, people with less formal education or from less affluent groups and those who are geographically isolated.

Despite the INCLUDE recommendations, developed via expert consensus, there remains a dearth of quantitative data regarding UK trial inclusivity. In 2022 the NIHR reviewed data reported in 148 NIHR funded randomised controlled trials conducted between 2007-2017 and published 2019–2021 [6]. Sex and ethnicity were compared with population level data from the 2011 England and Wales decennial census. Sex of participants matched proportions reported in the census (51% female, 49% male). Of the 60% of trials reporting ethnicity, the proportion of non-white participants was found to be broadly consistent with 2011 census data. The NIHR have recently released recommendations around data collection to improve monitoring of representation within research, covering all protected characteristics under the Equality Act, and others have recommended development of uniform standards to capture aspects of diversity, including language, religious practices and sexual orientation [5].

In our area of focus, oncology, we are aware of only one quantitative study into diversity, conducted at a single centre and published in 2010. This concentrated on ethnicity and compared admitted oncology patients with oncology trial participants, using data from 2000–2005 [7]. Ethnicity data were poorly recorded, however an analysis adjusted for disease, age and gender found that patients from minority ethnic groups had a lower chance of being in a research trial than white patients (Odds ratio (OR) = 0.70 (95% confidence interval (CI): 0.53 to 0.94); *p* = 0.01).

The large majority of published datasets demonstrating a lack of inclusion in trials use data from the United States. Although these give indications of potential areas for improvement in the UK, it is likely that patterns of participation differ due to the variations in historical, societal and demographic context, together with the universal coverage of the UK’s National Health Service—free at the point of use in contrast to the US’ private sector healthcare system.

Given the recommendations from INCLUDE, and findings in other research settings identifying systematic exclusion of some populations, [8] we conducted an audit to determine whether our trial protocols were explicitly excluding any under-served groups and our patient information provision was inclusive, and to assess whether we would be able to identify under-representation from the data we collect as standard.

## Methods

At the methodologist-led academic Clinical Trials and Statistics Unit at The Institute of Cancer Research (ICR-CTSU) we have designed, managed and analysed a substantial number of phase II and III oncology clinical trials including over 30,000 adult participants worldwide. We conduct investigator-initiated non-commercial trials funded by charities, government-funding schemes and pharmaceutical industry partners. Phase II trials generally focus on repurposing existing treatments with known safety profiles, we do not conduct trials for regulatory licensing purposes or on behalf of commercial sponsors. Interventions comprise radiotherapy, chemotherapy, hormone therapy and immunotherapy and disease sites include breast, prostate, bladder, head and neck, lung, and rare cancers.

All clinical trials managed by ICR-CTSU which gained UK regulatory approval between 2011 and 2022 and which were sponsored by either The Institute of Cancer Research or our partner organisation The Royal Marsden NHS Foundation Trust, were included in the audit. Trials with external sponsors were excluded to ensure audited trials had used ICR-CTSU templates and processes.

Documents were reviewed for a broad range of items which could be mapped onto the INCLUDE under-served groups (Table 1) [4].

**Table 1:** INCLUDE under-served groups mapped to items reviewed in the audit

	**Items reviewed in each document:**			
	**Protocol – eligibility criteria**	**Patient information material**	**Case report forms – data collection**	**Patient completed questionnaires – data collection**
**Demographic factors**				
Age extremes (e.g., under 18 and over 75)	Minimum/ maximum age	N/A	Date of birth	Date of birth
Women of childbearing age	Pregnancy status/ age/ sex	N/A	Date of birth / sex	N/A
Black, Asian and Ethnic Minorities	Ethnic group	N/A	Ethnic group	Ethnic group
Male/female sex (depending on trial context)	Sex/gender	Use of gendered words	Sex/gender	Sex/gender
LGBTQ + /sexual orientation	Sexual orientation	Use of gendered words	Sexual orientation	Sexual orientation
Educational disadvantage	N/A	Readability scores and word counts, availability in non-written format	Highest education level	Highest education level
**Social and economic factors**				
In full time employment	N/A	N/A	Employment status	Employment status collection
Unemployed/ low income^ab^	N/A	N/A	Employment status/ income	Employment status/ income
Military veterans	N/A	N/A	Military veteran status	Military veteran status
People in alternative residential circumstances (migrants, asylum seekers, prison populations, care homes, traveller communities and homeless)^ab^	N/A	N/A	Residence details	Residence details
People living in remote areas^a^	Geographical area/access to hospital	N/A	Residence details/ postcode	Residence details/ postcode
Religious minorities	N/A	N/A	Religion	Religion
Carers^a^	N/A	N/A	N/A	Carer status
People not fluent in the majority language	Fluent in English	Availability of patient information in alternative languages	Main language	Main language, availability in alternative languages
People who do not attend regular medical appointments	*Not possible to identify from trial documents*			
People in multiple excluded categories	*Not possible to identify from trial documents*			
Socially marginalised people^a^	*Not possible to identify directly – possible other groups in this category indicated with *^*a*^			
Stigmatised populations^b^	*Not possible to identify directly – possible other groups in this category indicated with *^*b*^			
Looked after children	*Not relevant to ICR-CTSU trials – all conducted in adult population*			
**Health status**				
Mental health conditions^ab^	Mental health condition exclusions	N/A	N/A	N/A
People who lack the capacity to consent for themselves^ab^	*Not relevant to ICR-CTSU trials – all require capacity to consent*			
Cognitive impairment^ab^	Brain metastasis	N/A	N/A	N/A
Learning disability^ab^	Learning disability	Availability in different formats	N/A	N/A
People with addictions^ab^	Substance addiction	N/A	Medical history collection	Medical history collection
Pregnant women	Pregnancy status	N/A	N/A	N/A
People with multiple health conditions^a^	HIV status / prior malignancies	N/A	Baseline symptoms/ medical history collection	Baseline symptoms/ medical history collection
Physical disabilities^a^	Physical accessibilty requirements /performance status	N/A	Baseline symptoms/ medical history collection	Baseline symptoms/ medical history collection
Visually/ hearing impaired^a^	N/A	Availability of documents in different formats	N/A	N/A
Too severely ill	Performance status	N/A	Baseline symptoms/ medical history/ performance status collection	Baseline symptoms/ medical history/ performance status collection
Smokers^b^	N/A	N/A	Smoking status collection	Smoking status collection
People living with obesity^b^	N/A	N/A	Weight collection	Weight collection
Rare diseases and genetic disease sub-types	N/A	N/A	Medical history collection	Medical history collection
People in cancer trials with brain metastases	Brain metastasis status	N/A	Medical history collection	Medical history collection

^a^Potentially socially marginalised groups

^b^Potentially stigmatised groups

For each trial meeting the selection criteria the first UK ethics approved version of the protocol, patient information sheet (PIS) and patient-completed questionnaire, together with the first version of case report forms (CRFs), were reviewed.

Where trials had multiple patient information sheets (PIS), these were categorised as being either comprehensive, summary, or for a substudy. The PIS using the most gendered terms was selected for review, regardless of category. To investigate accessibility for those with educational disadvantage, Flesch Kincaid grade level, the Flesch Reading Ease, word count, and average number of words per sentence were determined using Office 365 Microsoft Word’s inbuilt tools. Flesch-Kincaid grade level refers to US school year, ranging from 0–18, with a grade of 9 equivalent to a reading age of 13–14 [9]. Flesch reading ease ranges from 0–100, with a higher score indicating easier readability [10]. Where trials had more than one comprehensive PIS, a mean of the word counts and readability scores was calculated.

**Fig. 1:**
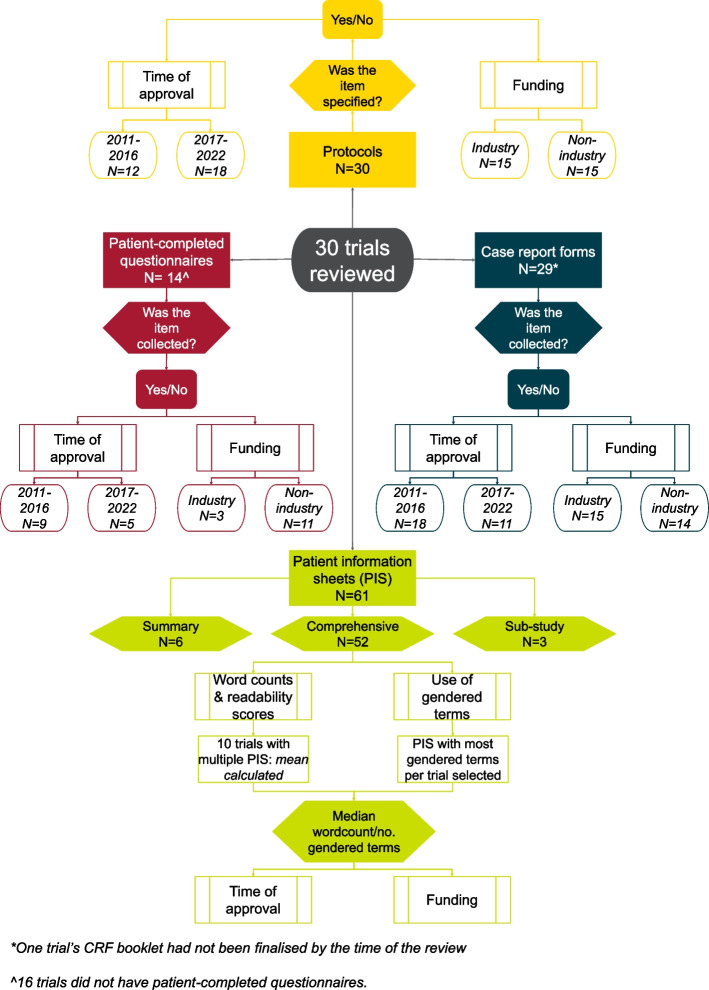
Audit process and trial categorisation

Audit data were tabulated for all trials and analysed combined and separately: 1) according to the time of trial approval (2011–2016 vs 2017–2022), to identify any trends or improvements in practice and 2) by funding source, according to receipt of any funding from industry partners—industry funded trials were the most likely to use potentially unlicensed agents or have eligibility requirements imposed by external partners (Fig. 1).

### Statistical methods

Analyses were conducted using Stata version 17. A *p*-value of < 0.01 was considered statistically significant to make some account for multiple testing. Fisher’s exact test was used to compare frequencies of items in protocols, CRFs and questionnaires by time of approval and funding source. For PIS review data, the median and quartiles were calculated for readability scores and word counts. Wilcoxon rank sum test was used to compare PIS review data by time of approval and funding source.

## Results

30 trials met the inclusion criteria for the audit conducted between March and May 2022 (Fig. 1). Trial characteristics are summarised in Table 2.

**Table 2:** Trial characteristics

	Date of approval				**Total**
	2011–2016		2017–2022		
Type of funding	Non-commercial funding	Industry funding	Non-commercial funding	Industry funding	
	**9**	**9**	**6**	**6**	**30**
Phase					
II	4	8	0	5	**17**
II/III	1	0	2	0	**3**
III	4	1	4	1	**10**
Trial type					
CTIMP	3	9	1	6	**19**
Non-CTIMP	6	0	5	0	**11**
Cancer type					
Breast	1	3	2	3	**9**
Multiple: breast, lung & prostate	1	0	0	0	**1**
Gynaecological	0	1	0	1	**2**
Head and neck	1	0	1	0	**2**
Lung	0	0	1	0	**1**
Penile	1	0	0	0	**1**
Prostate	1	4	2	1	**8**
Urinary system	4	1	0	1	**6**

### Demographic factors

90% (27/30) of protocols specified a lower age limit. 18/27 (67%) had a lower limit of 18, 8/27 (30%) had a lower limit of 16. One out of the 27 (3%) protocols specified a lower age limit of 60 years or that participants should be post-menopausal. Of the three trials that did not specify age, two were approved between 2017 and 2022 and had industry funding, the other was approved between 2011 and 2016, with non-commercial funding. There was no evidence of a difference according to time of trial approval or industry support. No protocol stated an upper age limit and all CRFs and patient-completed questionnaires collected date of birth.

No trial’s eligibility criteria specified ethnicity. Ethnic group was collected in 83% (24/29) of trials’ CRF booklets and was not captured in any patient-completed questionnaires.

Nine of the thirty trials reviewed were for cancers affecting people of male sex, seven included only female sex patients and the remaining 14 trials were open to all sexes. All trials enrolling people who could potentially become pregnant stated pregnancy was an exclusion for safety reasons. 14/30 (47%) protocols used a gendered term such as ‘women’, ‘men’, ‘male’, or ‘female’ in their eligibility criteria, none stated whether they referred to gender identity or biological sex. 50% (7/14) of trials using gendered terms included patients of only one sex due to the type and location of tumour (eg prostate cancer). The remaining seven protocols including gendered terms were for non-sex specific disease areas and stated both men and women could be included. There was no difference in use of gendered terms by the time of trial approval or type of funding.

83% (25/30) of patient information sheets used at least one gendered term (men, women, he, she). 23/30 (77%) trials used at least one gendered pronoun when referring to patients and 2/30 (7%) trials used gendered terms for clinicians only. Five trials did not include any gendered terms in patient information. 10/14 (71%) trials that were non-sex specific used a gendered term to address patients (Table 3).

**Table 3:** Use of gendered terms for patients in patient information sheets

	Male terms	Female terms	Male and female terms	No gendered terms	**N (*N* = 30)**
Male sex specific trial	6	0	0	3^a^	**9**
Female sex specific trial	0	7	0	0	**7**
Non-sex specific trial	0	1^b^	9	4^c^	**14**
Total	**6**	**8**	**9**	**7**	**30**

^*a*^*Two trials approved* > *6 years ago, of which one was industry funded and the other non-industry funded. The other trial was approved* < *6 years ago and industry funded*

^*b*^*Trial approved* < *6 years ago and industry funded*

^*c*^*Two trials approved* < *6 years ago and two approved* > *6 years ago. Three were industry funded and one was non-industry funded*

15/29 (52%) CRFs collected whether the participant was male or female, of which 12/15 captured sex and the remainder captured gender. Of the 12 trials capturing sex, one trial (2021 ethics approval) collected “sex at birth”. 12/14 (86%) trials that did not collect sex or gender were sex-specific due to the anatomical site of the tumour, three included female patients and nine male. There was no difference in collection of this information by the time of trial approval or by funding source.

Sexual orientation was not collected on any CRFs and neither sex/gender nor sexual orientation were collected in any patient-completed questionnaires.

**Fig. 2:**
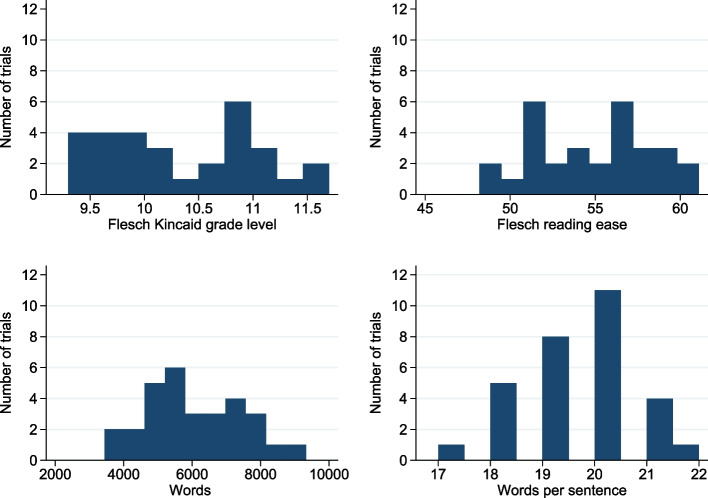
Patient information sheet readability scores and word counts

Average patient information sheet readability scores and word counts suggested that the median information sheet was suitable for people with a reading age of 15–16, with a median Flesch reading ease score of 55.6. Most trials’ information sheets had between 5500–6000 words and approximately 250 sentences (Fig. 2). PIS for trials approved between 2011–2016 had a higher Flesch reading ease score than that for trials approved between 2017–2022 (*p* = 0.003). No information was provided in a non-written format at time of first approvals and education level was not collected in any trial’s CRF but was collected in two questionnaires.

### Social and economic factors

Employment status was collected in one (3%) trial’s CRF and three trials’ (21%) questionnaires. One questionnaire also captured marital, childcare, and carer status. Religion was not collected in any trial’s CRFs. No CRFs collected income level, residence details or military status. Geographic location (postcode) was collected in 69% (20/29) of trial CRFs and 50% (7/14) of patient-completed questionnaires. No trials specified a language requirement, however the first version of all trials’ patient information sheet and patient-completed questionnaire was only available in English. Main language was not collected in any CRFs or patient questionnaires.

Ten percent (3/30) of trials had socio-geographic requirements, with an eligibility criterion stating: *“Absence of any psychological, familial, sociological or geographical condition potentially hampering compliance with the study protocol and follow-up schedule; those conditions should be discussed with the patient before registration in the trial.”* The time of trial approval or source of trial funding had no influence on whether this was included and no explanation was provided in the protocols.

There was no difference in any socio-economic factors being included in eligibility criteria or captured by CRFs or patient-completed questionnaires either by the time of trial approval or the funding type.

### Health status

**Fig. 3:**
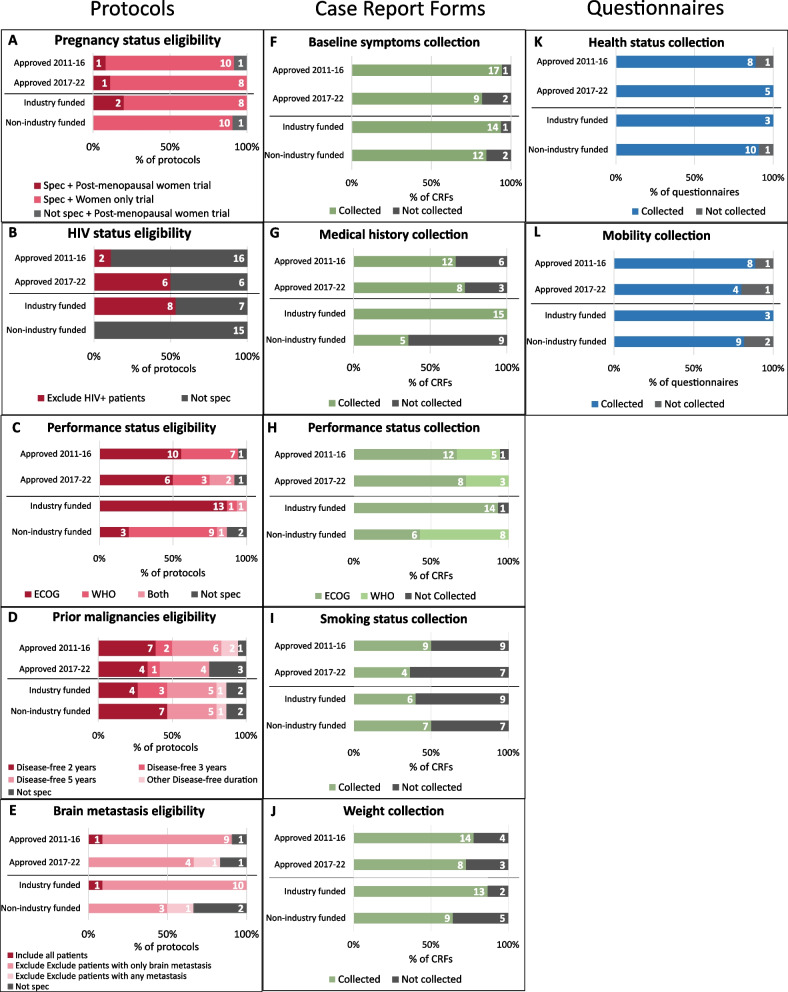
Health status eligibility criteria and data collection. *Abbreviations: Spec* = *specified. Not spec* = *not specified. ECOG* = *Eastern Cooperative Oncology Group; WHO* = *World Health Organisation A) Pregnancy status eligibility. Pregnancy status N* = *21, 9 trials excluded as they had only male sex participants. E) Brain metastasis N* = *17, 13 trials excluded as they were in early-stage non-metastatic cancer, so all metastases were excluded*

All protocols’ eligibility criteria included statements regarding non-permitted co-morbidities, which were well collected in CRFs and patient-completed questionnaires (Fig. 3). 87% (26/30) of protocols permitted inclusion of patients with prior malignancies, subject to a disease-free duration varying between 4–6 months to 5 years. Three trials’ protocols included a statement regarding psychological conditions, as noted above. No trials mentioned learning disability, substance addiction or physical requirements other than performance status. No trials provided information or patient questionnaires in formats accessible to people with visual impairments.

Time of approval did not have any impact on whether trials specified comorbidity exclusions or collected related details in CRFs or patient-completed questionnaires. Positive HIV status (Fig. 3) was the only co-morbidity in eligibility criteria found to be significantly different according to trial funding (*p* = 0.002), with all industry funded trials excluding people with HIV. A statistically significant difference was found between trial funding and medical history collection in CRFs (*p* =  < 0.001), with industry funded trials collecting medical history more frequently than non-commercially funded trials.

## Discussion

We found that our trial protocols did not have overt systematic exclusion for the majority of groups identified in the INCLUDE guidance. Our patient information generally included gendered terms and required a relatively high level of reading comprehension, with readability worsening for trials approved after 2016. Data collected for the purposes of assessing the trials’ endpoints, whether via CRF or patient completed questionnaire, was insufficient to identify groups suggested as under-served by research by INCLUDE. There was little difference in practice over time or by funding source.

Cancer is largely a disease affecting older people—of the 375,400 people diagnosed with cancer each year in the UK between 2016–2018, 194,500 were over 70 [11, 12]. Evidence from the United States suggests that older adults are under-represented in cancer trials [13]. Whilst it was encouraging that none of our protocols specified an upper age limit, we recognise that some co-morbidity exclusions, often required for safety when testing newer agents, may de-facto exclude some older people. Over 50% of those aged 65 and older have at least two chronic conditions and this is projected to increase in the future [14]. In addition, cancer incidence is strongly associated with a number of lifestyle choices, such as smoking, that are likely to increase burden of co-morbidities, [15] so it is crucial that co-morbidity exclusions are minimised when safe to do so. Patients with co-morbidities can be included in oncology trials when clinically appropriate, for example in later phase trials or academic phase II trials repurposing existing treatments with known safety profiles, to facilitate better representation of the population that the intervention is aimed towards.

Lower age limits were dictated by the UK regulatory landscape with its differing requirements for paediatric cancer trials. ICR-CTSU trials investigate cancers which are far more prevalent in older populations and are highly unlikely to affect younger people, therefore most of our clinical trials had 18 (age of UK adulthood) as their lower age limit. Some had a lower limit of 16, this is likely as a result of 2018 guidance from the UK’s National Cancer Research Institute’s Teenage, Young Adults and Germ Cell Tumours Group which recommended that Cancer Research UK (CRUK), a major funder of oncology trials in the UK, should request justification for lower age limits for studies they were supporting to avoid inadvertent exclusion of adolescents [16]. Drug effects are similar in adolescents and adults and by changing lower limits to 16 this would allow people to access new treatments earlier than otherwise possible, due to the tendency for paediatric trials to be conducted several years after trials in adults [17]. Despite this guidance, we did not see any association between time of trial approval or source of funding and lower age limit. This was likely due to trials being funded by other non-commercial funders, together with investigator consensus that changing the lower limit would be irrelevant due to the lack of incidence in younger populations.

Ethnicity was not stipulated in any inclusion criteria and was generally well collected in CRFs. This is encouraging as it should allow us to compare our trial participants’ data with UK cancer incidence statistics to identify any signals of under-representation in current practice. However we recognise that UK health records regarding ethnicity, from which the CRF data would likely be reported by hospital staff, are not always accurate particularly for those outside the White British category, [18] so we also intend to investigate direct collection from trial participants in future.

We identified a lack of clarity in our use of sex and gender nomenclature, both within protocols and in data collection. Whilst some trials were necessarily restricted to one sex due to the nature of the tumour, its location, or the intervention studied, around half were open to any sex. All trials enrolling people who could potentially become pregnant stated pregnancy was an exclusion. Whilst this is a group identified as under-served by research by INCLUDE, due to the nature of treatments studied in our trials it would be very difficult to justify loosening this criterion due to the danger of foetal exposure to cytotoxic agents, radiation or hormonal therapies.

All data collection regarding sex/gender used binary categories (male/female), which does not capture the known range of gender identities within the UK [19] and also does not recognise existence of intersex individuals, incidence of which is admittedly low, but too poorly measured in the UK to provide robust figures [20]. In addition there was frequent use of gendered terms both in protocols and patient information. Whilst we did not collect any data related to transgender identity, we recognise that people identifying as different genders to the sex they were assigned at birth may still be eligible for our single sex trials so patient information should ideally avoid use of gendered terms to avoid alienating people. Prostate cancer trials, for example, should include anyone with a prostate, such as some intersex or non-binary people and trans-women, as the prostate is not normally removed in gender affirming surgery [21]. A review of literature published between 1975 and 2017 identified only 10 published cases of transgender women with prostate cancer [22]. It is unlikely that incidence in this group is so low, but the findings may suggest an issue with data collection and reporting. As a result of our findings we have updated our templates and guidance to recommend the removal of gendered terms in protocols and CRFs wherever not required, and to be clear about whether they refer to sex or gender identity where they are used.

We did not collect any information related to participants’ sexual orientation, however LGBTQ+ people who have had cancer have shown a preference for gender-neutral language to address themselves and their partners [23]. We have therefore updated our patient information guidance to recommend removal of all gendered terms to prevent any inadvertent discouragement of participation of people from the LGBTQ+ community.

We assessed our patient information as a proxy for systematic exclusion of people with educational disadvantage and those who were not fluent in English or had visual impairments. In the UK, all medical research studies are expected to provide written information to potential participants, reviewed by Research Ethics Committees during the approval process [24]. There are no requirements to provide information in languages other than British English or in alternative formats, although recommendations to consider the latter have recently been introduced [25]. Thus it is unsurprising that we did not find any alternative formats or languages were available at first approval of our studies, although some trials did provide short versions of the full patient information sheet. We are currently exploring the best approach to providing information in alternative languages, about which there is very little guidance for researchers in the UK, and introducing guidance around use of validated non-English language patient-completed questionnaires. We are also beginning to introduce audio-visual presentation of clinical trial information for selected trials as this may improve patient understanding, although this remains a topic requiring further research [26].

It was disappointing to observe a trend towards lower readability of patient information over time. This may be associated with an increased level of complexity for more recent trials, although this was not formally reviewed within this audit. Approximately 15% of UK adults had literacy levels of 9–11 years or younger in 2011, representing an estimated 5.1 million people [27]. Our patient information sheets, with a median reading age of 15–16, are therefore likely to be too complex for a large proportion of the UK population despite routine involvement of patient and public representatives in their development. This is consistent with other research in the UK and Republic of Ireland finding the median reading age for information sheets to be 16.1 years, with a median Flesch Reading Ease score of 49.6 [28]. We are seeking to improve readability by implementing more training in the use of plain English and recommending use of Word readability statistics whilst preparing patient information. In addition, whilst there used to be a suggested template provided by the NHS Health Research Authority including mandatory sections which contributed to the overall length of information provided, current advice recommends reducing length of information to be *“enough to make an informed choice about taking part, and no more” *[25]. It is therefore likely that length of our patient information sheets could be reduced in future, and we hope that readability will improve, both as a result of process improvements implemented as a result of this study and due to the changing landscape in ethics review processes.

Cancer incidence is higher in socio-economically disadvantaged populations, [29] and according to data from North America, patients from these backgrounds are less likely to take part in oncology trials [30]. However there are few recent published data investigating the impact of socio-economic status on research participation in the UK. Despite having universal healthcare coverage via the NHS, this does not mean that trial participation is without cost, as it may involve additional visits necessitating more time off work and leading to higher transport costs than associated with standard of care.

Our relatively routine collection of participants’ postcodes will allow identification of those living in remote areas, together with some indication of inclusivity via the Indices of Multiple Deprivation (IMD) tools, available for each devolved UK nation [31]. However the IMD is not a reliable measure for individuals’ level of deprivation, as within each area people’s circumstances will vary. Without collecting social and economic factors directly from participants it is not possible to robustly assess the majority of such groups identified within INCLUDE from our trial datasets.

Reassuringly, the majority of protocols did not include any statements regarding social and economic factors, or overtly exclude patients in the majority of under-served health status groups proposed by INCLUDE. However, three trial protocols did include statements regarding patients from certain socio-geographical backgrounds. These could result in people being less likely to be invited, or to decide not to join these trials based on clinicians’ preconceptions regarding their ability to comply and resulting challenging conversations. We were unable to identify why such a statement had been included as there was no discernible pattern and have updated our protocol guidance to ensure these are not included in future. Excluding populations from remote geographical locations may be due to the locations of specialist cancer treatment centres, however, we are currently improving guidance on obtaining support to allow patients to be enrolled at geographically-distant sites across the country and to travel for the intervention. This approach is being taken in the TORPEDO trial [32] to avoid excluding patients due to location, but requires buy-in from non-commercial funders who have historically been hesitant to fund participants’ travel and/or accommodation costs due to restricted budgets.

Health status was well collected in CRFs, and patient-completed questionnaires. The majority of co-morbidities and medical history requirements listed in protocols appeared directly related to safety requirements for the interventions under study, with an association between funding source and HIV status likely to be due to industry funded trials using newer agents than those in non-commercially funded trials. Participants’ history of other cancers, with a disease-free duration prior to trial entry, was often specified, however the required disease-free duration ranged from a few months to a few years. This did not appear to be associated with the stage of cancer being investigated. Prior cancer disease-free duration could be shorter for trials in the metastatic setting, where endpoint events such as cancer progression or death are unfortunately likely to be reached within a short time after enrolment. In earlier stage disease, participants may need to be followed up for many years and this could risk a recurrence of their prior cancer being conflated with a recurrence of the cancer being studied within the trial. However, in our audit, trials in the metastatic setting did not routinely stipulate a shorter cancer-free period than trials for patients with early-stage disease. A more systematic approach for deciding the disease-free period for prior cancers in trial eligibility criteria is a potential area for improvement in our protocols.

Inclusion of patients with a high burden of comorbid conditions, with likely associated use of multiple medications, may mean that the effects of these cannot be reliably disentangled from symptoms caused by the trial treatment or impact on survival outcomes. Concomitant medication may also interfere with the intervention’s mechanism, leading to exclusions for safety purposes. However blanket exclusion of a high number of co-morbidities and concomitant medications could lead to overestimating the safety of an intervention before it is rolled out to the wider population, so it is critical to strike the right balance between protecting participants safety and ensuring inclusion of a representative group of patients.

This audit had limitations as we could only consider factors over which we have influence, including the design of our trials, development of research protocols and patient information and data capture practices. We reviewed the first-approved version of trial documents—later versions of documents may have been more inclusive, although we saw few discernible patterns when we looked at later approved trials in comparison to earlier ones. The audit does not account for other barriers to inclusion that are not possible to identify from trials’ essential documents and data capture alone. We have not systematically collected sufficient data to allow assessment of all under-served groups, as this information is not needed to assess any of the reviewed trials’ outcomes. We have historically taken the approach of focused data collection for the purposes of ensuring protocol adherence, safety and endpoint analysis, both to avoid collecting data that would not be used, which would not be ethically justified, and to avoid over-burdening NHS hospitals with unnecessary data collection.

As our trial participants are not identified and recruited by ICR-CTSU, but by clinicians and their research staff within participating NHS hospitals, we would require a different dataset to ensure robust unbiased assessment of equitable inclusion. We are planning to access national healthcare datasets to explore this further.

Despite its limitations, this audit represents a starting point for our planned programme of work investigating inclusivity in UK oncology trials and has identified several areas for improvement to our current practice as described above. We plan to work with patient and public contributors of differing backgrounds and life experiences to determine an acceptable level of enhanced demographic data collection, informed by the NIHR’s recent recommendations. We recognise we need to collect enough information to better monitor representation in future trials whilst balancing proportionality of potentially intrusive data collection and ensuring acceptability to participants.

There is a distinct lack of published quantitative data regarding inclusivity in UK cancer trials, across all protected characteristics and under-served groups, which makes any impact of process improvements difficult to discern. Obtaining and publishing these data is a key area of focus in our future research plans.

## Conclusion

Our trials’ eligibility criteria were relatively inclusive. Data were routinely collected regarding co-morbidities, age, ethnic group, and sex/gender. Other demographic and social and economic factors were not frequently collected. Process improvements implemented as a result of this audit, such as use of gender neutral terminology, recommendations around minimising co-morbidity exclusions where possible, and considering collecting more demographic factors, may also be relevant to other academic trial groups.

## Data Availability

The datasets used and/or analysed during the current study are available from the corresponding author on reasonable request.

## Abbreviations

CI: Confidence interval
CRFs: Case report forms
CTIMP: Clinical Trial of Investigational Medicinal Product
HRA: NHS Health Research Authority
ICR-CTSU: Clinical Trials and Statistics Unit at The Institute of Cancer Research
INCLUDE: Innovations in Clinical Trial Design and Delivery for the Under-served
MHRA: Medicines and Healthcare products Regulatory Agency
NHS: National Health Service
NIH: National Institutes of Health
NIHR: National Institute for Health and Care Research
PIS: Patient information sheet
UK: United Kingdom
US: United States of America
